# Arsenite exposure suppresses adipogenesis, mitochondrial biogenesis and thermogenesis via autophagy inhibition in brown adipose tissue

**DOI:** 10.1038/s41598-019-50965-9

**Published:** 2019-10-08

**Authors:** Jiyoung Bae, Yura Jang, Heejeong Kim, Kalika Mahato, Cameron Schaecher, Isaac M. Kim, Eunju Kim, Seung-Hyun Ro

**Affiliations:** 1 Department of Biochemistry, University of Nebraska, Lincoln, NE 68588 USA; 2 Department of Cell and Regenerative Biology, University of Wisconsin School of Medicine and Public Health, Madison, WI 53705 USA; 3 Department of Neurology, The Johns Hopkins University School of Medicine, Baltimore, MD 21205 USA; 4 College of Medicine, University of Nebraska Medical Center, Omaha, NE 68198 USA; 5 Department of Mechanical and Materials Engineering, University of Nebraska, Lincoln, NE 68588 USA

**Keywords:** Mechanisms of disease, Diseases

## Abstract

Arsenite, a trivalent form of arsenic, is an element that occurs naturally in the environment. Humans are exposed to high dose of arsenite through consuming arsenite-contaminated drinking water and food, and the arsenite can accumulate in the human tissues. Arsenite induces oxidative stress, which is linked to metabolic disorders such as obesity and diabetes. Brown adipocytes dissipating energy as heat have emerging roles for obesity treatment and prevention. Therefore, understanding the pathophysiological role of brown adipocytes can provide effective strategies delineating the link between arsenite exposure and metabolic disorders. Our study revealed that arsenite significantly reduced differentiation of murine brown adipocytes and mitochondrial biogenesis and respiration, leading to attenuated thermogenesis via decreasing UCP1 expression. Oral administration of arsenite in mice resulted in heavy accumulation in brown adipose tissue and suppression of lipogenesis, mitochondrial biogenesis and thermogenesis. Mechanistically, arsenite exposure significantly inhibited autophagy necessary for homeostasis of brown adipose tissue through suppression of Sestrin2 and ULK1. These results clearly confirm the emerging mechanisms underlying the implications of arsenite exposure in metabolic disorders.

## Introduction

Obesity is a significant risk factor for several prevalent diseases, such as diabetes, hypertension, cardiovascular diseases, and cancers^1–4^. White adipose tissue (WAT) and brown adipose tissue (BAT) are the main types of adipose tissue in humans^5^. In contrast to WAT’s functions for energy storage, BAT is specialized to produce ATP through enriched intracellular mitochondria and heat through non-shivering thermogenesis via the uncoupling protein 1 (UCP1)^6–9^. UCP1 proteins are localized in the inner membrane of brown adipocyte mitochondria to uncouple ATP synthesis from respiration^10–13^. While BAT quickly regresses following birth^5,14^, new evidence has revealed symmetrical fat depots in adults that have classic BAT features^15–18^. Therefore, BAT has become a novel target for obesity treatment and prevention.

Arsenite, a most potent trivalent form of arsenic, is presented in water, soil, and foods due to its abundance in earth crust and the use of arsenite-contaminated pesticides and insecticides^19^. Arsenite is accumulated in various tissues and organs including adipose tissues, lung, heart, kidney, brain, eye, liver, hair, bone, and spleen^20–22^. Thus, arsenite contaminated drinking water has been considered as a contributing factor for numerous health concerns in humans such as diabetes, lung and skin diseases, and cancer^23–25^. Recent evidence suggests that exposure to arsenite may lead to adipose tissue dysfunction and lipodystrophy as well as inhibition of adipogenesis^26,27^. Arsenite inhibits adipogenesis and adipocyte function in human mesenchymal stem cells^28^, 3T3-L1 preadipocytes^29^, and C3H 10T1/2 preadipocytes^30^. The mechanisms underlying adipose tissue dysfunction and inhibition of adipogenesis by arsenite have been the focus of intense research. Recent studies suggest that arsenite-induced lipolysis is mediated through transcriptional factors, including peroxisome proliferator-activated receptor-gamma (PPARγ) and CCAAT-enhancer binding protein alpha (C/EBPα)^28,29^, as well as β-adrenergic receptor signaling^31^.

Autophagy is a delicate cellular recycling process in response to environmental and genotoxic stresses, which digests damaged proteins and organelles as a defense mechanism^32^. Mechanistically known so far, AMPK positively regulates autophagy induction through phosphorylating and activating ULK1, mammalian homologue of autophagy kinase Atg1, at Ser317, Ser555, and Ser777 whereas mTORC1 inhibits autophagy induction through phosphorylating and inhibiting ULK1 at Ser757^33–36^. ULK1 regulates downstream autophagosome receptors and autophagosome maturation proteins such as p62^37^ and LC3B, a mammalian homologue of autophagy-related gene (Atg) 8^38^. Recent studies have shown that autophagy plays an important role in 3T3-L1 adipogenesis and lipid metabolism. Autophagy deficient 3T3-L1 preadipocytes block differentiation into mature adipocytes^39^. Moreover, Atg5 or Atg7 deficient primary mouse embryonic fibroblasts (MEF) impair adipogenesis, and autophagy inhibitor, such as bafilomycin A1 and chloroquine, blocks primary MEF differentiation^40,41^.

Despite arsenite-induced defect in adipogenesis and fat accumulation in WAT, the roles of arsenite in regulating the functions of BAT remain to be elucidated. Here, we report the impacts of arsenite exposure on the functions and activities of brown adipocytes and BAT. In cultured brown adipocytes, arsenite treatment reduced adipogenesis, mitochondrial biogenesis, respiration and thermogenesis. Arsenite exposure in live mice resulted in heavy arsenic accumulation in BAT but not in WAT. Accumulated arsenite suppressed lipogenesis, mitochondrial biogenesis and thermogenesis in BAT. Autophagy activity was significantly reduced by arsenite exposure through inhibition of Sestrin2 and ULK1. Our data provides the novel mechanisms underlying the effects of arsenite exposure on the physiological functions of BAT and reveals the significance of autophagy in protection against arsenite-induced damage in BAT.

## Results

### Arsenite suppresses HIB1B brown adipocyte differentiation

**Figure 1:**
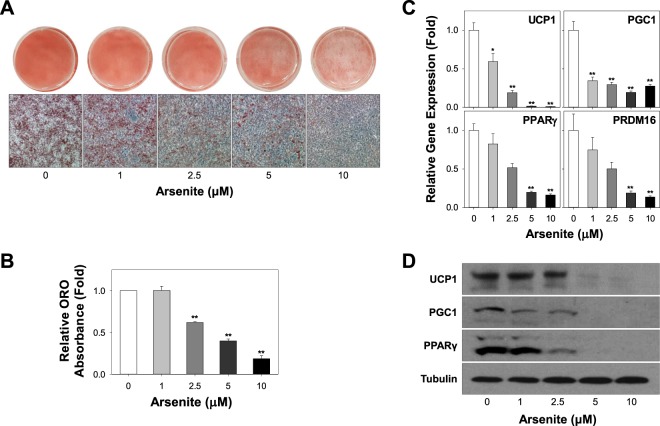
Arsenite exposure suppresses differentiation of HIB1B brown preadipocytes. (**A**) HIB1B brown preadipocytes were differentiated in the presence of increasing doses of arsenite (1–10 μM) or the vehicle control. (**B**) Oil Red O (ORO) staining was performed for cell morphology and ORO absorbance at Day 6 (D6) was measured. (**C, D**) After the differentiation of brown preadipocytes in the presence of increasing doses of Arsenite (1–10 μM) at Day 6, mRNA and protein expression of established brown markers were analyzed by qRT-PCR (**C**) and immunoblotting (**D**), respectively. At least 9 independent experiments were performed and all data was expressed as mean ± SEM. *P < 0.05 or **P < 0.01 by One-way ANOVA analysis.

To investigate the effects of arsenite on brown adipocyte differentiation, immortalized HIB1B brown preadipocytes were differentiated in the presence of various doses of arsenite (1–10 μM) or the vehicle control. Arsenite dose-dependently suppressed brown adipocyte lipid accumulation, as revealed by Oil Red O stained multiocular, lipid-filled brown adipocytes (Fig. 1A). 10 μM of arsenite treatment decreased brown adipocyte differentiation upto 82% (Fig. 1B). Upto 10 μM of arsenite did not affect cell viability confirmed by MTT assays for upto 6 days (Fig. S1). mRNA expression of brown adipocyte specific markers, uncoupling protein 1 (UCP1), peroxisome proliferator-activated receptor-gamma coactivator 1 (PGC1), peroxisome proliferator activator receptor gamma (PPARγ), and PR domain containing 16 (PRDM16) (*P* < 0.01) was significantly decreased by arsenite treatment (Fig. 1C). Attenuated protein expression of UCP1, PGC1, and PPARγ (Fig. 1D) was consistent with decreased mRNA expression levels, suggesting that arsenite negatively affect differentiation of HIB1B brown preadipocytes by downregulating expression levels of essential brown adipocyte specific markers in both gene and protein.

### Arsenite suppresses mitochondrial biogenesis and respiration in HIB1B brown adipocytes

**Figure 2:**
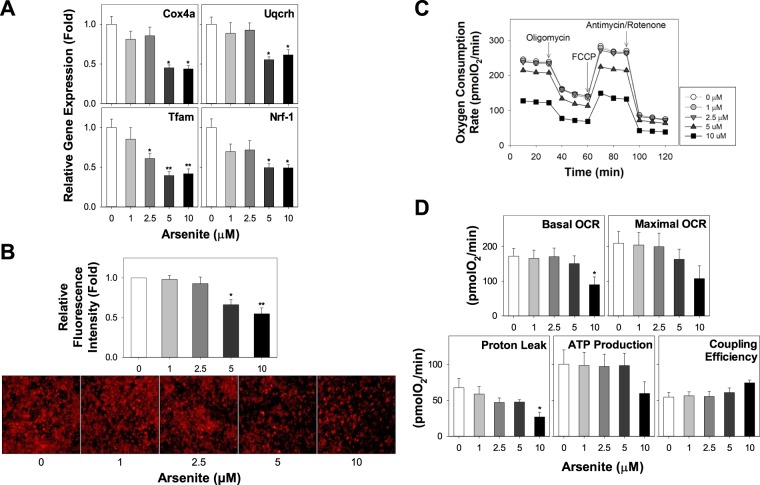
Arsenite dose-dependently suppresses mitochondria-associated marker genes, content and mitochondrial respiration in brown adipocytes. (**A**) Brown preadipocytes were differentiated in the presence of increasing doses of arsenite (1–10 μM) or the vehicle control. mRNA expression of mitochondrial structural genes, Cox4a, and Uqcrh, and transcriptional factors, Tfam and Nrf-1, at Day 6 (D6) was measured. (**B**) Mitochondrial content was analyzed by mitochondrial-specific fluorescence dye MitoTracker at Day 6. Relative fluorescence intensity was calculated from the mean fluorescence intensity values of the samples and expressed as fold of the control. (**C**) Brown preadipocytes were seeded in 24-well XF assay plates and differentiated in the presence of increasing doses of arsenite (1–10 μM) or the vehicle control until Day 6. The cells were subjected to real-time measurements of oxygen consumption rate. At least 9 independent experiments were performed and all data was expressed as mean ± SEM. *P < 0.05 or **P < 0.01 by One-way ANOVA analysis.

Analysis of mRNA expression showed that arsenite also suppressed mRNA levels of nucleus-encoded mitochondrial genes, cytochrome c oxidase subunit IV a (Cox4a) and cytochrome b-c1 complex subunit 6 (Uqcrh), and transcription factors regulating mitochondrial gene expression, transcription factor A (Tfam) and nuclear respiratory factor 1 (Nrf-1) (Fig. 2A). Consistent with the mRNA expression results, mitochondrial biogenesis demonstrated by the staining of mitochondrial specific fluorescence MitoTracker Red was also decreased by arsenite treatment (Fig. 2B). To determine whether the suppression of mitochondria biogenesis by arsenite leads to decrease in mitochondrial uncoupling and thermogenesis, we examined oxygen consumption rates coupled with mitochondrial stress tests in brown adipocytes which have been differentiated in the presence or absence of arsenite by cellular bioenergetics measurements using XF24 Extracellular Flux Analyzers. Arsenite at 10 μM significantly decreased basal oxygen consumption rate (OCR) and proton leak; however, arsenite did not affect the maximal OCR, ATP production, and coupling efficiency (Fig. 2C,D).

### Autophagy activity was inhibited by arsenite treatment during brown adipocyte differentiation

**Figure 3:**
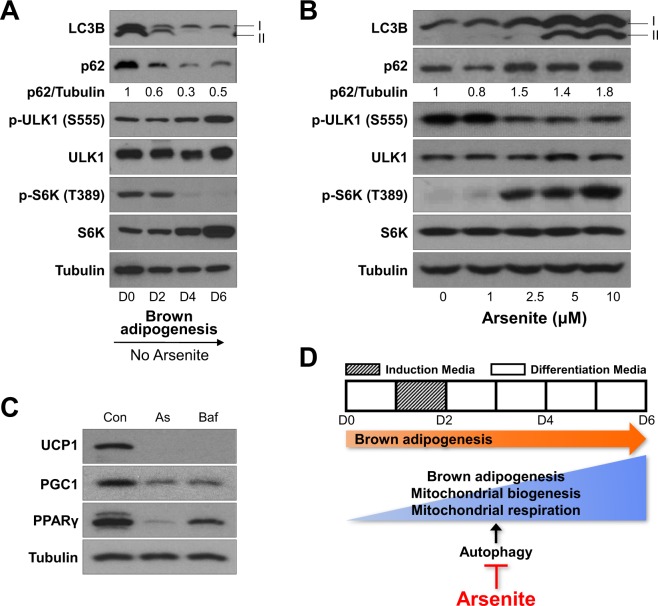
Arsenite inhibits autophagy which is necessary during brown adipocytes differentiation. (**A**) HIB1B brown adipocytes were differentiated without arsenite treatment and whole cell lysates at Day 0, 2, 4, and 6 (D0, 2, 4, and 6) were analyzed for autophagy activity. (**B**) Brown preadipocytes were differentiated in the presence of increasing doses of arsenite (1–10 μM) or the vehicle control until Day 6 (D6). Whole cell lysate was prepared and analyzed by immunoblotting with specific antibodies. (**C**) Arsenite (10 μM), Bafilomycin A1 (0.1 µM) or the vehicle control was treated during the differentiation of brown adipocytes and the protein levels of differentiation markers were measured by immunoblotting. (**D**) Schematic diagram depicting the toxic effect of arsenite on brown adipogenesis via inhibition of mitochondria function, thermogenesis and autophagy. At least 9 independent experiments were performed.

We examined whether autophagy signaling underlies brown adipocyte differentiation, immunoblotting analysis showed that autophagy activity was up-regulated during brown adipocyte differentiation consistent with previous report in 3T3-L1 white adipocyte differentiation^42^. Autophagosome marker, microtubule-associated protein 1 light chain 3-II (LC3B-II, mammalian homologue of Atg8) and autophagosome substrate p62 (aka sequestosome 1) were both degraded by active autophagolysosome upto 50% by 10 µM arsenite treatment, phosphorylation of unc-51 like autophagy activating kinase 1 (ULK1) at Ser 555, which is AMPK target site, was increased and phosphorylation of ribosomal protein S6 kinase (S6K) at Thr 389, which is mTORC1 target site, was decreased upon brown adipocyte differentiation process (Fig. 3A). We also confirmed that arsenite suppressed autophagy induction during brown adipocytes differentiation revealed by accumulation of both LC3B-II and p62 upto 1.6 times in 10 μM of arsenite, decrease of ULK1 activity (p-Ser 555) and increase of S6K phosphorylation (p-Thr 389) when the cells were treated with arsenite in dose dependent manner (Fig. 3B). We examined the effect of the autophagy inhibitor, bafilomycine A1, on expression of brown adipocyte specific markers. Bafilomycine A1 significantly attenuated the expression of UCP1, PGC1, and PPARγ protein expression levels in brown adipocytes (Fig. 3C), suggesting that autophagy pathway is necessary for brown adipogenesis, and supporting our hypothesis that arsenite suppresses brown adipocyte differentiation and function via autophagy inhibition through ULK1 inhibition and mTORC1 activation (Fig. 3D).

### Arsenite was heavily accumulated in brown adipose tissue of mice after oral administration

**Figure 4:**
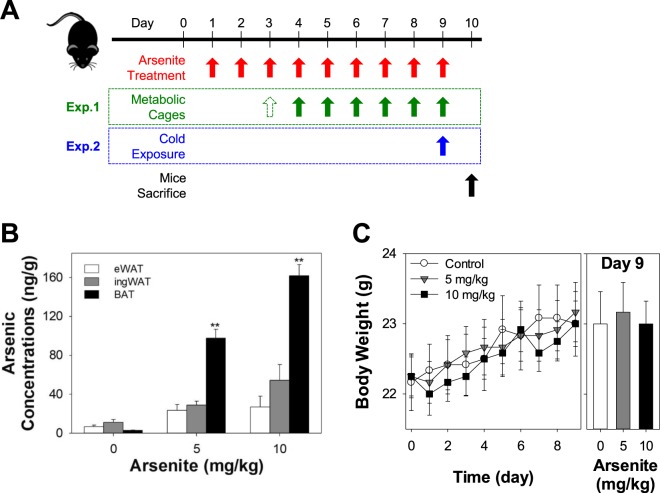
Arsenite is heavily accumulated in brown adipose tissue (BAT) but not in white adipose tissue (WAT) of mice. (**A**) Experimental design for mouse work. (**B**) The arsenic accumulation was quantified in eWAT, ingWAT, and BAT of C57BL/6 mouse using ICP-MS after the oral administration of arsenite at doses of 0 (control), 5, and 10 mg/kg/day for 9 days (*n* = 6). Adipose tissues of all mice were collected on Day 10 under anesthesia with isoflurane and CO_2_. (**C**) Measurement of body weights (*n* = 12). All data was expressed as mean ± SEM. *P < 0.05 or **P < 0.01 by One-way ANOVA analysis.

Knowing that arsenite treatment suppresses the differentiation and mitochondrial function of mouse brown adipocytes in dose dependent manner (Figs 1 and 2), we investigated whether arsenite can be accumulated on mouse BAT in physiological condition. First, the arsenic accumulation was quantified in mouse BAT and WAT using the inductively coupled plasma mass spectrometry (ICP-MS) after administration of arsenite by oral gavage at doses of 5 and 10 mg/kg once a day for 9 days (Fig. 4A,B). The control group was given water at the same volume of arsenite treatment groups. The relative amount of arsenite in BAT and WAT of those mice was compared. The concentration of arsenic accumulated in BAT was 4.5 times and 7.9 times higher than that in WAT (*P* < 0.01) in mice that were treated with 5 mg/kg and 10 mg/kg treatment groups, respectively. Although previous report identified that arsenic can majorly be accumulated in liver, kidney, and lung^19,25^, we never expected such a high and differential accumulation between BAT and WAT in mice. Given that the accumulated concentration of arsenic in BAT but not in WAT was increased in dose-dependent manner, we anticipated that some of physiological parameters of mouse would be affected by arsenite accumulation in BAT. However, we could not observe any significant differences in body weight (Fig. 4C) and food and water intakes (Fig. S2A) between control and arsenite treatment groups. We also did not observe any significant differences in oxygen consumption (VO_2_) and respiratory exchange ratio (RER, VO_2_/VCO_2_) when normalized by mouse body weight (Fig. S2B,C).

### Arsenite down regulates the gene expression of brown adipose markers *in vivo*

**Figure 5:**
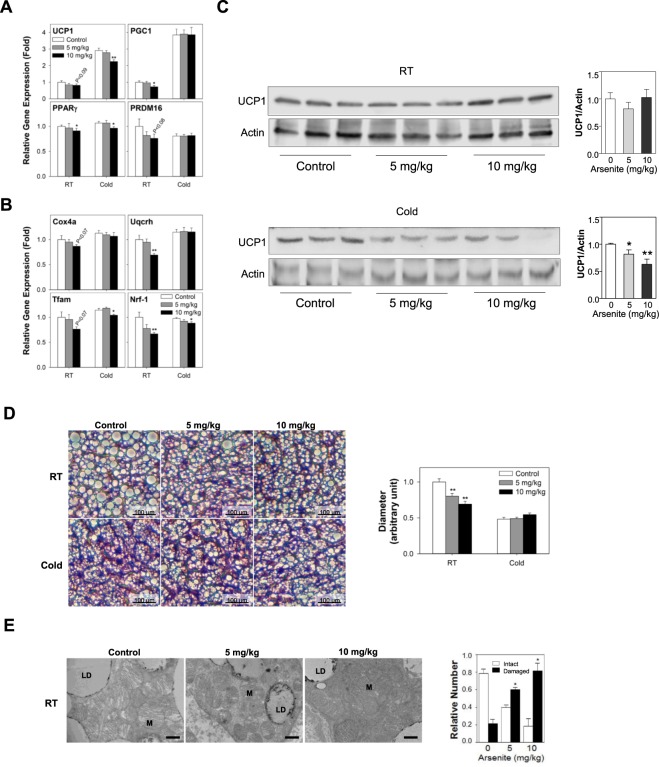
Arsenite down regulates the gene expression of brown adipocyte markers including thermogenesis gene UCP1 of mouse BAT *in vivo*. (**A**,**B**) mRNA expression levels of BAT markers (**A**) and mitochondrial biogenesis genes (**B**) were analyzed by qRT-PCR after the oral administration of arsenite into mice at doses of 0 (control), 5, and 10 mg/kg/day for 9 days. Mice were challenged with room temp (RT, 23 °C) control or cold temp (Cold, 4 °C) for 24 hrs on the last day of oral gavage to evaluate thermogenesis function (*n* = 6). (**C**) Protein expression level of UCP1 from mice BAT after administered with 0 (control), 5, and 10 mg/kg/day arsenite for 9 days (RT) and exposed to cold temp for 24 hrs before euthanization was analyzed by immunoblotting (*n* = 3). Relative band intensities were quantified through densitometry (ImageJ, NIH). (**D**) H&E staining of mouse BAT after arsenite administration and quantitative analysis of lipid droplet size (*n* = 6). Black scale bars represent 100 μm. (**E**) TEM image of mouse BAT after arsenite administration and quantitative analysis of relative number of intact and damaged mitochondria (*n* = 3). LD, lipid droplet, M, mitochondria. Scale bar, 800 nm. All data was expressed as mean ± SEM. *P < 0.05 or **P < 0.01 by One-way ANOVA analysis.

To evaluate the effects of accumulated arsenite on BAT homeostasis in mice, we measured mRNA expression of brown adipose specific markers, UCP1, PGC1, PPARγ, and PRDM16, after the oral administration of arsenite at doses of 5 and 10 mg/kg/day for 9 days (Fig. 5A). Lipogenic gene, PGC1 and PPARγ, expression levels of 10 mg/kg group were significantly down regulated compared to those of control group (*P* < 0.05). The mRNA expression of nucleus-encoded mitochondrial biogenesis genes, Cox4a, Uqcrh, Tfam, and Nrf-1, were also measured on mice BAT (Fig. 5B). Uqcrh and Nrf-1 gene expression levels of 10 mg/kg group were significantly decreased compared to those of control group (*P* < 0.01). These data suggest that arsenite accumulated in 10 mg/kg group effectively suppresses brown adipocyte function and mitochondrial biogenesis. A well-known function of BAT is non-shivering thermogenesis that has been spotlighted in the regulation of obesity pandemic^43,44^. Since UCP1 expression is augmented when exposed to cold in BAT of mice^45^, we hypothesized that UCP1 inhibition by arsenite would be more dramatic and significant because UCP1 is further induced under by cold exposure. To investigate the effects of arsenite on UCP1 expression in non-shivering thermogenesis in mouse BAT, we challenged the mice in cold condition (4 °C) for 24 hrs at the last day of arsenite administration (Fig. 4A). Arsenite administration (10 mg/kg) significantly reduced UCP1 (*P* < 0.01) and PPARγ (*P* < 0.05) gene expression levels while there was no significant difference in PGC1 and PRDM16 gene expression levels (Fig. 5A). In the case of genes involved in mitochondrial biogenesis, Tfam and Nrf-1 gene expression levels of 10 mg/kg group were significantly reduced compared to those of control group (*P* < 0.05) whereas Cox4a and Uqcrh had no significant difference (Fig. 5B). Consistent with the gene expression level of UCP1, protein expression level of UCP1 was also suppressed by arsenite exposure in cold condition in mice BAT (Fig. 5C). Combined together, these data suggest that arsenite (10 mg/kg for 9 days) may suppress thermogenesis through the inhibition of UCP1 expression. Consistent with the suppression of adipogenesis genes such as PGC1 and PPARγ, arsenite decreased the lipid droplet (LD) diameter of BAT in mice at room temperature (23 °C). However, in the BAT of mice exposed to cold (4 °C) for 24 hrs at the last day of arsenite administration, LD diameter was not significantly changed regardless of arsenite doses (Fig. 5D) possibly due to no significant change in PGC1 and PRDM16. Nevertheless, the overall reduction of lipid content under cold temperature relative to room temperature would reflect the induction in thermogenesis by activating UCP1 in BAT. The UCP1 inhibition by arsenite could not be enough to observe a significant increase in LD diameter. Transmission electron microscopy (TEM) image reveals that there was 3 and 4 fold increase in the number of either sick (e.g. missing cristae structure) or broken (e.g. ruptured outer membrane structure) mitochondria in BAT collected from mice in 5 and 10 mg/kg arsenite-administered groups, respectively (Fig. 5E). Total BAT mass was significantly reduced in the group of 10 mg/kg arsenite administration (Fig. S3) due to the decrease in adipogenesis and LD droplet size and mitochondrial biogenesis.

### Arsenite blocks autophagy activity through Sestrin2 and ULK1 inhibition *in vivo*

**Figure 6:**
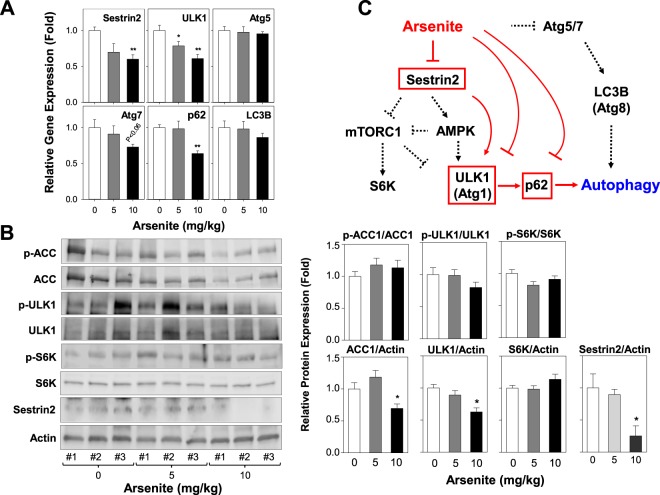
Arsenite inhibits autophagy in mouse BAT. (**A**) mRNA expression levels of autophagy genes in mouse BAT were analyzed after the oral administration of arsenite into mice at doses of 0 (control), 5, and 10 mg/kg/day for 9 days. (**B**) Protein expression levels of autophagy pathway genes were measured by immunoblotting in mouse BAT (*n* = 3) after arsenite administration. Relative phosphorylation band intensities were quantified through densitometry (ImageJ, NIH) and presented as mean ± SEM (*n* = 3). *P < 0.05 by One-way ANOVA analysis. (**C**) Proposed model of how arsenite suppresses autophagy activity in BAT. Arsenite administration suppresses autophagy mainly through direct inhibition of Sestrin2 and ULK1. Arsenite suppresses gene expressions of p62 so that autophagosome receptor binding is blocked in mouse BAT. Arsenite has no significant effect on Atg5- or Atg7-dependent autophagosome conjugation system.

It was reported that short term treatment or exposure of arsenite increases reactive oxygen species (ROS) level and induces autophagy as a defense mechanism in human uro-epithelial cells^46^. To investigate the effect of arsenite treatment on autophagy activity in BAT, we measured mRNA expression of essential autophagy genes (e.g., ULK1, Atg5, Atg7, and LC3B), stress-inducible protein Sestrin2, and autophagy receptor p62 under our experimental conditions. Sestrin2, ULK1, and p62 were significantly downregulated by arsenite (10 mg/kg BW of mice) (Fig. 6A) suggesting that arsenite may inhibit autophagy by downregulating genes involved in ULK1 induction as well as p62 receptor association with autophagosome. Sestrin2 inhibits mTORC1 through AMPK activation^47,48^. Therefore, arsenite may inhibit gene expression of Sestrin2 to lead to mTORC1 activation through AMPK inhibition and subsequent autophagy inhibition. Although our immunoblotting data displayed a significance decrease in Sestrin2 and ULK1 expression levels in response to arsenite administration (10 mg/kg), there was no significant difference in both phosphorylation of S6K (a mTORC1 target) at Thr 389 and phosphorylation of ACC (an AMPK target) at Ser 79 (Fig. 6B). These data collectively suggest that the autophagy inhibition by arsenite is mainly attributed to the inhibition of Sestrin2 and ULK1 expression but not through direct mTORC1 or AMPK regulation. While arsenite may directly inhibit autophagosome maturation by inhibiting p62, there was no significant effect of arsenite on the expression of genes involved in the Atg5-, Atg7-, or LC3B- dependent autophagosome conjugation system (Fig. 6C).

## Discussion

Increased consumption of high carbohydrate and fat containing food and westernized diet would have been a serious concern correlated with the increased rate of morbidity due to metabolic diseases such as obesity, diabetes, cardiovascular diseases, and cancer. Particularly combined with the exposure to harmful environmental pollutants and genotoxic agents in the food, water and air, it would have been major attention in combating against metabolic diseases in the modern society^19,49^. United States environmental protection agency (EPA) classified arsenic as a Group 1A carcinogen. Over decades, majority of studies have focused on arsenic carcinogenesis in skin, lung, liver, and urinary bladder and the increased level of oxidative stress, mitochondria DNA damage, mitochondrial biogenesis, and mutation in arsenic sensitive oncogenes were believed to be major contributing factors in human^50^. Unlike arsenic carcinogenesis studies, the studies on the effect of arsenic on the adipose tissue metabolism, mitochondrial function in obesity were very limited albeit the positive correlation between arsenite exposure and diabetes mellitus in Michigan residing human studies^25^, and population studies in high arsenic areas (≥150 µg/L drinking water) of Taiwan and Bangladesh^51^, and the molecular mechanism underlying the arsenic-induced metabolic disorders is still remained unclear.

Other arsenic research groups reported that acute high dose of arsenic (>500 nM) increases ROS and subsequently upregulates antioxidant genes such as heme oxygenase and Nrf2 to protect cells from mitochondria damage and oxidative DNA damage^52^. However, low dose of arsenic (<500 nM) upto 24 hrs treatment decreases ROS by inducing antioxidant genes^53^. Furthermore, low dose of arsenic promotes cell proliferation by increasing mitochondrial biogenesis in skin cancer cells^54^. It is still debatable but generally ROS-mediated cell damage and apoptosis would be considered as mechanism underlying arsenic-induced toxicity. Our data suggest that upto 10 μM of arsenite treatment did not affect the cell survival without apoptosis induction, so we investigated the effect of arsenite treatment during the course of brown adipocyte differentiation. Arsenite treatment suppressed differentiation and inhibited mitochondrial biogenesis of brown adipocytes in a dose dependent manner. This data was consistent with the inhibition of white adipocyte differentiation in previous studies^29,30^. However, we observed 7.9 times more accumulation BAT compared to eWAT in 10 mg/kg administration of arsenite in mice. This data would suggest that arsenite-associated obesity is not only affected by inhibition of WAT function and is further aggravated by the malfunction of BAT *in vivo*. It needs further investigation why the arsenic is much heavily accumulated in BAT among adipose tissues in mice. It is possible that arsenic directly bind to the promoters of genes involved in the mitochondrial biogenesis or blocking mitochondrial turnover through inhibiting autophagy gene expression or autophagy protein activity by direct binding. Our group and others would investigate the individual arsenic specific target genes and its inhibition mechanism as further studies. High level of accumulation of arsenic prevent normal cell differentiation of adipose tissues and cause the imbalance in fat metabolism and mitochondrial functions, which can contribute to the development of metabolic disorders such as obesity and diabetes. ACC gene expression essential for lipid biogenesis was also suppressed in arsenite-administered mice group. Consistently, we have observed decrease in the lipid droplet content in BAT upon arsenite administration. Another notable result is that arsenite treatment suppresses UCP1 gene expression in the mitochondria, which are essential for thermogenic function of brown fat to dissipate energy as a heat^55^. In live mice, we observed similar result while total food and water intake, oxygen consumption and RER were not changed. It is possible that there would be compensation mechanism from other tissues protecting mice since oxygen consumption and RER are vital signs essential for survival. Transgenic overexpression of mitochondrial uncoupling protein UCP1-3 in skin of mice showed strong resistance to chemically induced carcinogenesis, suggesting that inhibition of mitochondrial respiration through enhancing mitochondrial uncoupling would be a therapeutic target against arsenic-induced cancer^56^. In the same context, UCP1 overexpression or UCP1 enhancing drugs would be a potential treatment to protect adipose tissues from arsenic-induced metabolic disorders. It is plausible that proper dose of arsenic chelating agents such as BAL, DMPS, and DMSA^57^ or overexpressing arsenic-sequestrating proteins such as Hexokinase-2 (HK2)^58^ in BAT would be beneficial in improving lipid metabolism, mitochondrial function and thermogenesis, which contribute on the prevention or treatment of arsenic-induced metabolic disorders.

Short term treatment upto 48 hrs or acute exposure of arsenite induces autophagy evidenced by increasing lipidated form of LC3B (LC3B-II) and decreasing level of p62 autophagy receptor in cardiac cells, neuronal cells, and cancer cells^59–61^. Arsenite-induced ROS in short term treatment have been suggested as a main cause to induced autophagy activity^62^. However, in recent studies using NIH3T3 fibroblasts, 4 hrs of short term treatment of arsenite activates Nrf2 pathway through autophagy inhibition^63^. Indeed, in our autophagy studies using HIB1B brown adipocytes, we observed increased level of LC3B-II accumulation along with the accumulation of p62 by arsenite, which support the inhibition of autophagy pathway. Treatment of autophagy blocker, bafilomycin A1 also completely suppress the genes of brown adipogenesis, which also support the inhibition of brown adipocyte differentiation via autophagy inhibition by arsenite. It has been reported by several independent groups that a loss of essential autophagy genes such as Atg5 and Atg7 leads to the formation of p62-positive aggregates and accumulation of ubiquitinated protein inclusion body and Mallory bodies in the liver^64–66^. Autophagy has two LC3B or Atg8 conjugations systems, Atg5-Atg12^67^ and Atg4-Atg7-Atg3^68,69^. However, our data showed that arsenite mediated autophagy inhibition in BAT is independent of Atg5 and Atg7 (*P* < 0.06). Rather, Sestrin2, ULK1 and p62 inhibition by arsenite accumulation is the underlying mechanism of autophagy inhibition in BAT. Recently, Gao *et al*. reported that arsenite treatment upto 10 μM for short term 24 hrs blocks autophagy flux through LncRNA UCA1 mediated activation of mTORC1/S6K pathway in human hepatocytes^70^. However, in our studies, arsenite treatment through oral gavage over longer term 9 days in mice suppressed Sestrin2 and ULK1 gene expressions but not significantly inhibiting AMPK and activating mTORC1. Ro *et al*. previously reported that Sestrin2 can directly bind with ULK1 and activate autophagy induction possibly independently from mTORC1 and AMPK^71^. Longer arsenite exposure would directly regulates gene expression levels of Sestrin2 and ULK1 to inhibit autophagy. Differently from cell culture model, there might be a compensation mechanism that maintains mTORC1 or AMPK activity under arsenite-induced toxicity in mammals. Our TEM image data also suggested that inhibition of autophagy activity in arsenite treatment groups may contribute on the accumulation of damaged mitochondria in BAT. Autophagy inhibition by arsenite exposure would lead to irreversible apoptotic cell death which we observed in the reduction of total BAT mass including both mitochondria and lipid mass by arsenite. In this regard, autophagy enhancing drugs such as metformin, torins and resveratrol^72^ or the overexpression of autophagy induction proteins such as Sestrin2 and ULK1 would be protective mechanism against deregulation or loss of BAT functions affected by arsenic-induced toxicity. While our group is preparing the manuscript, two recent papers came out on publication. Consistent with our data suggesting the autophagy inhibition by arsenite, Dodson *et al*. from Dr. Zhang’s group treated As (III) concentrations ranging 0.5 to 5 µM [37.5 to 375 ppb] in NIH 3T3 fibroblasts upto 48 hrs and observed that arsenic inhibits SNARE complex formation by enhancing O-GlcNAcylation of SNAP29 and drives autophagy dysfunction^73^. However, in another arsenic research group suggested that arsenic at 50 ppm (equivalent of 50,000 ppb or 667 µM) in drinking water administration in NMRI mice under 20 weeks of HFD actually increased both macroautophagy and Chaperon-mediated autophagy (CMA) in liver tissues^74^. Since the macroautophagy response is more rapid and the CMA is activated when macroautophagy is inhibited or defective under prolonged nutritional stress^75–77^, the upregulation of gene expression levels of both macroautophagy and CMA genes would be a compensation effect of serious macroautophagy or CMA defect and possible non-apoptotic cell death in liver. From these recent publications, we speculate that the arsenic effect on autophagy function is highly debatable depending on arsenic concentrations, time, tissue types, and mice species. Therefore, our study has proposed a unique mechanism and is an independent research from liver studies regarding the long term arsenic effect on BAT function and autophagy.

In conclusion, we propose a model where arsenite exposure consistently suppresses the brown adipocyte differentiation, mitochondrial biogenesis and function, thermogenesis, and autophagy function, which would have harmful effect on metabolic balance in human body and leads to metabolic disorders. Our study supports the previous human studies performed in US and other developing countries in the world that have higher concentration of arsenite contamination in water and soil would develop higher chances of development of metabolic disorders such as obesity and diabetes^23–25^. We believe that our study provides new clues on the connections between arsenite exposure and metabolic disorders by highlighting the major metabolic functions of BAT, which seemingly has higher accumulation of arsenic in our body. Balancing the BAT differentiation, mitochondrial integrity, thermogenesis, and autophagy activity would be novel protective mechanisms against arsenic-induced toxicity and metabolic disorders.

## Methods

### Reagents and antibodies

Dulbecco’s modified Eagle’s medium (DMEM) was purchased from Invitrogen (San Diego, CA). Fetal bovine serum (FBS), 3-isobutyl-L-methylxanthine, T3, dexamethasone, insulin, indomethacin, and Oil Red O were purchased from Sigma-Aldrich (St. Louis, MO). MTT cell growth assay kit was purchased from Sigma-Aldrich (St. Louis, MO). Primary antibodies against PPARγ, phospho-AMPK (T172), phospho-ULK1 (Ser555), ULK1, phospho-ACC (Ser79), ACC, and S6K were purchased from Cell Signaling Technology (Danvers, MA). Tubulin and Actin antibodies was purchased from Developmental Studies Hybridoma Bank (Iowa City, IA). PGC1α, phospho-S6K (Thr389), and AMPK antibodies were purchased from Santa Cruz Biotechnology (Dallas, TX) and UCP1 antibody was purchased from Alpha Diagnostic (San Antonio, TX). Sodium arsenite (NaAsO2) was obtained from Sigma-Aldrich (St. Louis, MO) and a stock solution was prepared at a final concentration of 100 mM and was diluted for a working concentration in water (ddH_2_O).

### Brown preadipocyte culture and adipocyte differentiation

Immortalized HIB1B murine brown preadipocyte cell line, generated from the interscapular brown fat of C57BL/6 mice, is a gift from Dr. Johannes Klein (University of Lubeck, Lubeck, Germany)^78^. Brown preadipocytes were maintained in DMEM supplemented with 20% FBS at 37 °C humidified atmosphere of 5% CO_2_ in air. The differentiation of brown preadipocytes was as previously described^79^. Briefly, cells were plated and grown in DMEM supplemented with 20% FBS until confluency (designed as D0). The cells were then changed into DMEM supplemented with 20% FBS, 1 nM T3, 20 nM insulin, 0.125 mM indomethacin, 5 μM dexamethasone, and 0.5 mM 3-isobutyl-L-methylxanthine for 24 hrs, followed by DMEM supplemented with 20% FBS, 1 nM T3, and 20 nM insulin, replenished every two days until the cells were fully differentiated at Day 6 (D6). Sodium arsenite was added to the media at D0 and replaced with each change of the media during the process. All experiments were performed in room temperature (23 °C) unless it is specifically indicated as in cold temperature (4 °C).

### Oil red O staining and quantification

Fully differentiated brown adipocytes were fixed with 10% formalin overnight, and the lipid droplets were stained with Oil Red O and quantified by absorbance at 500 nm in a spectrophotometer.

### Immunoblotting analysis

Total cell lysates were prepared in RIPA buffer (50 mM Tris-Cl pH 7.4, 150 mM NaCl, 1% sodium deoxycholate, 1% NP-40; 0.1% SDS) or cell lysis buffer (20 mM Tris-Cl pH 7.5, 150 mM NaCl, 1 mM EDTA, 1 mM EGTA, 2.5 mM sodium pyrophosphate, 1 mM β-glycerophosphate, 1 mM Na_3_VO_4_, 1% Triton-X-100) containing protease inhibitor cocktail (Roche) and phosphatase inhibitor (Sigma) and protein concentrations were determined by BCA assay kit (Thermo Scientific, Waltham, MA). Thirty micrograms of total cell lysates were subjected to 4–20% SDS-PAGE and transferred to polyvinylidene difluoride membrane. The membrane was blocked in 20 mM Tris-HCl, 137 mM NaCl, and 0.2% Tween 20 (pH 7.6) containing 5% nonfat milk. The membrane was immunoblotted with primary antibodies at 4 °C overnight followed by secondary antibody conjugated with horseradish peroxidase (Bio-Rad, Hercules, CA) for 1 hr. The membrane was exposed on X-ray film using ECL Western blot detection reagents (Pierce Biotechnology, Rockford, IL) or digitally captured using Odyssey blot imager (LI-COR, Lincoln, NE). Raw uncropped images with molecular size marker were displayed in the supplementary section (Figs S4–S9). Immunoblot band density was measured by ImageJ program (National Institute of Health) and normalized by the intensity of loading control (Tubulin or Actin) as indicated.

### RNA preparation and quantitative real-time PCR analysis

Total RNA was prepared using TRIzol (Invitrogen). Total RNA abundance was quantified using a NanoDrop Spectrophotometer (NanoDrop Technologies, Wilmington, DE). Reverse transcription was carried out using High Capacity cDNA Reverse Transcription kit (Thermo Scientific, Pittsburgh, PA) according to the manufacturer’s instructions. mRNA expression of target genes and housekeeping gene (Table S1) were measured quantitatively using SsoAdvanced Universal SYBR Green Supermix (Bio-Rad, Hercules, CA). PCR reactions were run in 96-well plate using MyiQ Real-Time PCR Detection System (Bio-Rad, Hercules, CA). Cycle conditions were 95 °C for 5 min, then 40 cycles of 95 °C for 15 sec/60 °C for 30 sec. Relative gene expression was calculated using the 2^−ΔΔ^Ct method, which normalizes against loading control β-actin.

### Analysis of mitochondrial content by MitoTracker staining

Mitochondria were labeled using the mitochondria-specific dye MitoTracker Red (Invitrogen, Carlsbad, CA) according to manufacturer’s protocol. Briefly, the differentiated brown adipocytes were washed and incubated with 100 nM MitoTracker Red for 30 min at 37 °C. Image was taken with Leica DMI4000B fluorescence microscope (Leica Microsystems, Wetzlar, Germany). The fluorescence intensity was measured with Synergy H1 Hybrid Multi-Mode Reader (BioTek Instruments, Winoosk, VT). Relative fluorescence intensity is the fold of the mean fluorescence intensity of the controls.

### Mitochondrial respiration measurements

Five thousand of brown preadipocytes were seeded in gelatin-coated 24-well XF assay plates in the 10% FBS containing DMEM and differentiated in the presence or absence of arsenite for 6 days. Differentiated brown adipocytes were rinsed 3 times, changed to 500 µL of XF assay buffer, and equilibrated at non-CO_2_ incubator at 37 °C for 1 hr. The cells subjected to real-time measurements of oxygen consumption rate (OCR) using XF24 Extracellular Flux Analyzer (Agilent Seahorse, Santa Clara, CA) as previously reported^45,55^. For mitochondrial stress tests, mitochondrial complex inhibitors were injected to all of the following treatments sequentially in the following order: oligomycine (1 µM), carbonyl cyanide-ptrifluoromethoxyphenylhydrazone (FCCP; 0.75 µM), antimycine A/rotenone (2.5 µM each), and 3 readings were taken after each injection. OCR was automatically recorded by XF24 software v2.3 provided by the manufacturer. Calculations of proton leak, coupling efficiency, maximal respiration, and ATP production were performed according to the manufacturer’s instructions.

### Animal handling

Housing and use of mice in the study were performed in compliance with the National Institute of Health (NIH) Principles for the Use of Animals, the American Veterinary Medical Association (AVMA) Guidelines, and the Public Health Service Policy on Humane Care and Use of Laboratory Animals awarded by University of Nebraska-Lincoln (Animal Welfare Assurance Number: A3459-01). Animal care and experimental procedures were approved by the institutional Animal Care and Use Committee (IACUC Approval Number: 1310 and 1527). Seven-week-old male C57BL/6J mice (Stock No. 000664, 21–23 g) were purchased from Jackson Laboratory (Bar Harbor, ME)^80–82^. They were maintained for a week to acclimate to their new environment before performing the experiments. Animals were housed in a group of three or four mice per cage (501 cm^2^ floor area; Tecniplast, Italy) with Tek-Fresh bedding (Harlan Teklad, Madison, WI) under a 12-h light/dark cycle from 06:00 to 18:00. They were fed the standard chow diet (Catalog #2016C, Envigo, Madison, WI) contains16% protein and 4% fat to cover 12% of total kcal and sterilized drinking water *ad libitum*. Mice were euthanized according to IACUC protocol after the completion of experiments.

### Oral administration of arsenite in mice and tissue collection

Sodium arsenite stock solution at 200 mg/mL was prepared by dissolving in distilled regular water. The arsenite stock solution was aliquoted and stored in −80 °C until use. The arsenite was orally administered to mice at doses of 5 and 10 mg/kg after diluting the arsenite stock solution with distilled water (*n* = 6 per group). A control group was given regular water at a dosing volume of 5 mL/kg which was comparable to the volume of arsenite administered groups^83,84^. The arsenite was administered to mice once a day for 9 days (Day 1 to 9)^85–88^. In the case of *in vivo*, we wanted to quantify the accumulated amount of arsenic in mice adipose tissues. The major source of arsenic exposure is drinking water and food. The exposure ordinarily occurs chronically. To ensure all mice ingested equal arsenic, we decided to administer arsenite to mice orally by gavage rather than including it in drinking water and food. The arsenic dose and treatment period were determined by tissue distribution and toxicity in consideration of the previous reports^83–88^. To check the levels of mRNA in BAT and to compare the change of them under different temperature, mice were divided into room temperature (RT, 23 °C) or cold temperature (4 °C). The mice under cold condition were transferred to cold room (4 °C) on Day 9 for 24 hrs. The body weight of mice was measured from Day 0 to 10. The epididymal white adipose tissue (eWAT), inguinal white adipose tissue (ingWAT), and interscapular brown adipose tissue (BAT) of all mice were collected on Day 10 under anesthesia with isoflurane and CO_2_. All samples were measured individually and stored at −80 °C until use. All experiments were performed in RT unless it is specifically indicated as in cold temperature.

### Quantification of arsenic content in adipose tissue

Arsenic analysis was performed using an Inductively Coupled Plasma Mass Spectrometry (ICP-MS) 7500cx series (Agilent Technologies, Santa Clara, CA) and an ESI SC-4 high-throughput auto sampler (Elemental Scientific, Omaha, NE). All mice adipose tissue (eWAT, ingWAT or BAT) samples were homogenized in PBS containing Complete Protease Inhibitor Cocktail (Roche, Basel, Switzerland). To normalize the lysate samples, the amount of protein in each lysate was measured using the Bradford Protein Assay (Bio-Rad, Hercules, CA). The adipose tissue samples were suspended in 200 µL of nitric acid with 50 µg/L of gallium as an internal standard. For complete digestion of fat, WAT samples were incubated overnight with 200 µL of nitric acid and internal standard followed by adding 200 µL of H_2_O_2_ and incubated for 15 hrs. The suspended adipose tissue samples were diluted 1–20 times into the auto sampler plate to analysis the arsenic content. Each sample was analyzed 3 times. The carrier solution was 1.5% nitric acid at a flow rate of 70 mL/min and the injection volume was 70 μL.

### Transmission electron microscopy (TEM) imaging

Brown adipocyte tissue was extracted from mice and reduced to three 1-mm pieces. Samples were fixed with 2.5% glutaraldehyde in 0.1 M cacodylate buffer (pH 7.4) for 1 hr at RT and then overnight at 4 °C. Samples were then post-fixed with 1% osmium tetroxide for 1 hr at RT. Fixed tissues were dehydrated through a graduated ethanol series and embedded In Spurr (Electron Microscopic Sciences, Fort Washington, PA). Ultrathin sections (~80 nm) were stained with uranyl acetate and lead citrate and observed under a transmission electron microscope (Hitachi H7500-I). A series of ultrastructural images were collected with a bottom-mount digital camera.

### Measurement of food and water intake and mitochondrial respiratory rate

The food and water intakes and respiratory rate of mice were measured using metabolic cages (TSE systems, Chesterfield, MO) during arsenite treatment period (*n* = 4 per group). The metabolic profiles were investigated by a computer controlled monitoring systems of metabolic cages as previously reported^45^. Mice were housed in groups before they were in the metabolic cages (Day 0 to 2) and then acclimated in metabolic cages, one mouse per cage, for a day (Day 3). They also were in the metabolic cages alone while the food and water intakes and respiratory rate were measured for 5 days (Day 4 to 9).

### Histology

A portion of the collected BAT samples was prepared for histology. Theses tissues were placed in cassettes and fixed in 10% formalin. All tissues were processed on a Leica Peloris tissue processor (Leica Biosystems, Wetzlar, Germany). The prepared sections (4 microns) were stained with haematoxylin and eosin (StatLab, McKinney, TX). Tissue slides were evaluated by light microscopy at 400X magnification. Six microphotographs per mouse were obtained. The lipid-droplet diameter of BAT tissues was calculated from the mean of ten values per microphotograph.

### Statistical analysis

All data are presented as mean ± SEM. Measurements were performed at least in triplicates. Statistical analysis was performed using SPSS 25 (IBM Coperation, Armonk, NY). One-way ANOVA was performed by Tukey’s multiple comparison test to determine the differences of group mean among control and two treatment groups. The level of significance was set at *P < 0.05 or **P < 0.01.

## Supplementary information

Supplementary Info
