# Supplementary material for: Arsenite exposure suppresses adipogenesis, mitochondrial biogenesis and thermogenesis via autophagy inhibition in brown adipose tissue
Source: Sci Rep. 2019 Oct 8;9:14464. doi: 10.1038/s41598-019-50965-9 (PMC6783448; doi:10.1038/s41598-019-50965-9)
Supplement: Supplementary file 1 — Supplementary Info [file 41598_2019_50965_MOESM1_ESM.pdf]

Supplementary information for:

Arsenite exposure suppresses adipogenesis, mitochondrial biogenesis and thermogenesis via autophagy inhibition in brown adipose tissue

Jiyoung Bae[1,2,+], Yura Jang[1,3,+], Heejeong Kim[1,+], Kalika Mahato[1], Cameron Schaecher[1,4], Isaac M. Kim[1], Eunju Kim[5] & Seung-Hyun Ro[1,*]

[1]Department of Biochemistry, University of Nebraska, Lincoln, NE, 68588, USA

[2]Department of Cell and Regenerative Biology, University of Wisconsin School of Medicine and Public Health, Madison, WI, 53707, USA

[3]Department of Neurology, The Johns Hopkins University School of Medicine, Baltimore, MD, 21205, USA

[4]College of Medicine, University of Nebraska Medical Center, Omaha, NE, 68198, USA

[5]Department of Mechanical and Materials Engineering, University of Nebraska, Lincoln, NE, 68588, USA

[+]These authors contributed equally to this work.

[*] Correspondence and requests for materials should be addressed to S-H.R (email: shro@unl.edu).

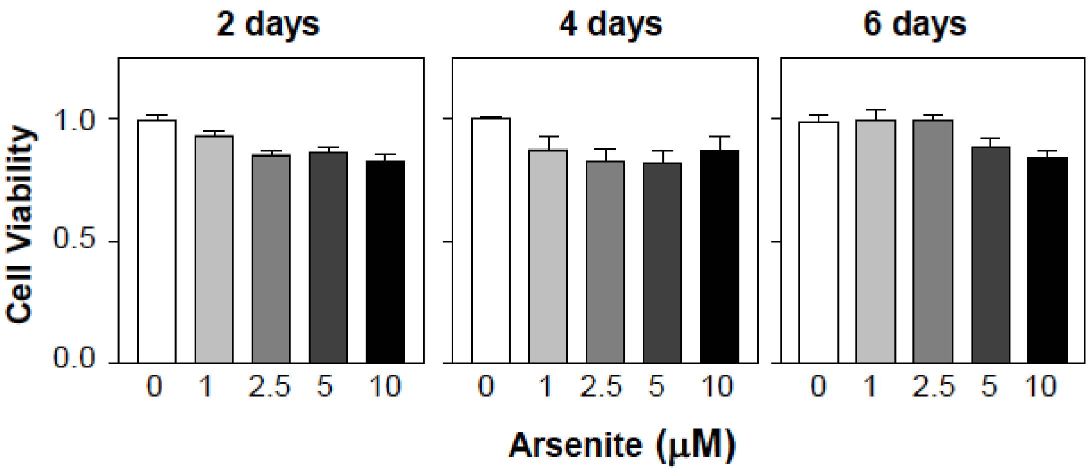

Figure S1. Arsenite has no significant effect on cell viability of HIB1B brown preadipocytes. MTT assay for cell viability measurement was performed in HIB1B brown preadipocytes after arsenite (1–10 μM) treatment for 2, 4 and 6 days. All data was expressed as mean±SEM by One-way ANOVA analysis.

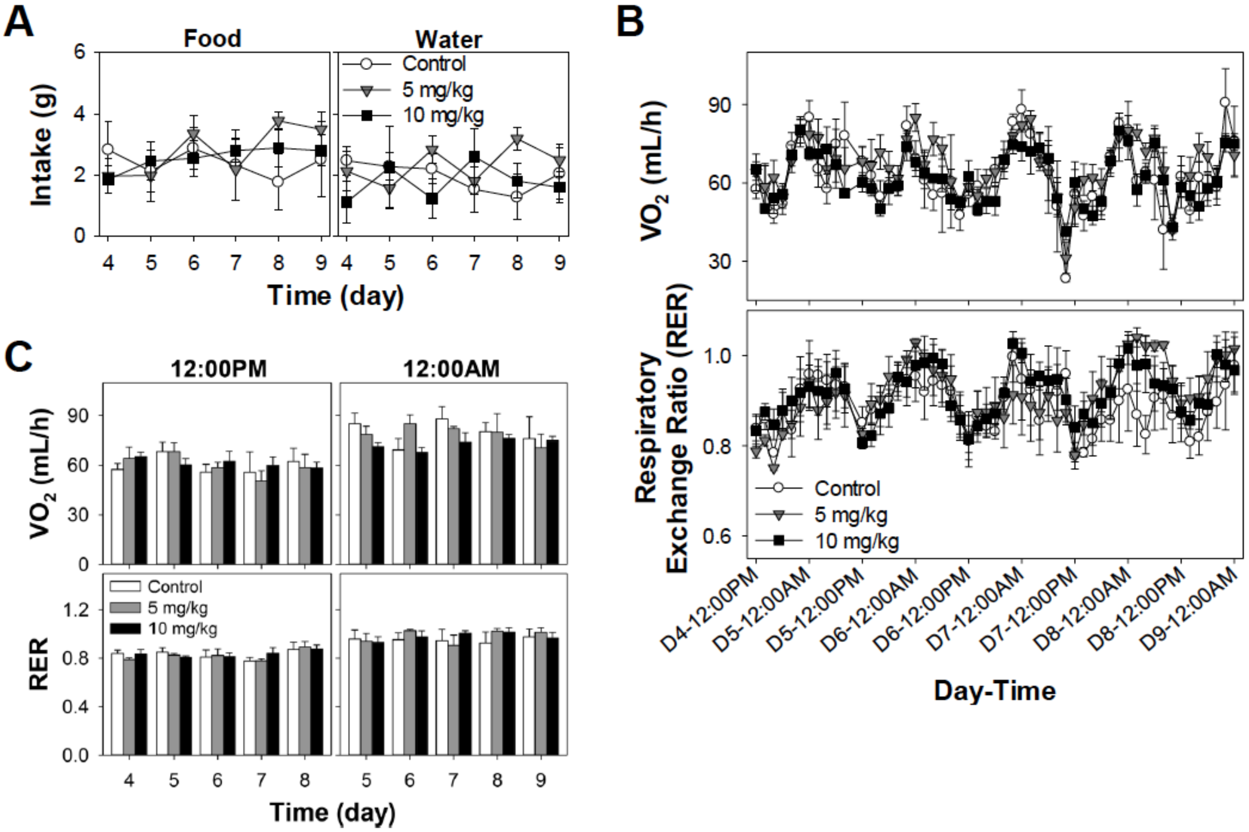

Figure S2. Arsenite does not significantly affect food and water intake and mitochondrial respiratory rate in mice BAT. (A) Food and Water intakes, **(B, C)** Oxygen consumption and RER were measured during arsenite administration (*n* = 6). All data was expressed as mean±SEM by One-way ANOVA analysis.

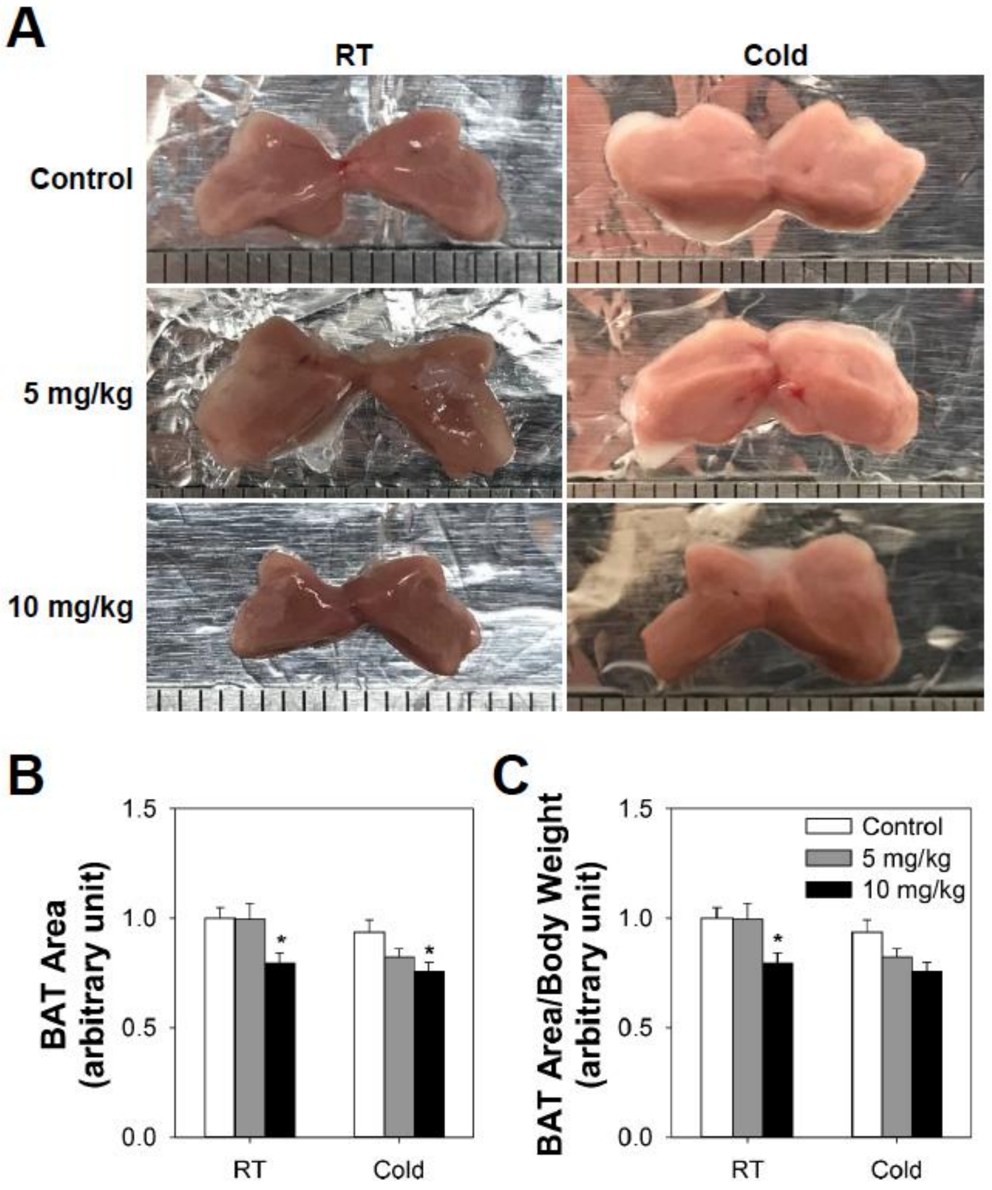

Figure S3. Arsenite exposure decreases overall BAT size and mass in mice. (A) Gross image of iBAT after the oral administration of arsenite into mice at doses of 0 (control), 5, and 10 mg/kg/day for 9 days. Mice were challenged with room temp (RT) control or cold temp (Cold, 4 °C) for 24 hrs on the last day of oral gavage. **(B)** BAT area and **(C)** BAT area normalized by body weight were quantitated ($n = 6$). All data was expressed as mean±SEM. $P^* < 0.05$ by One-way ANOVA analysis.

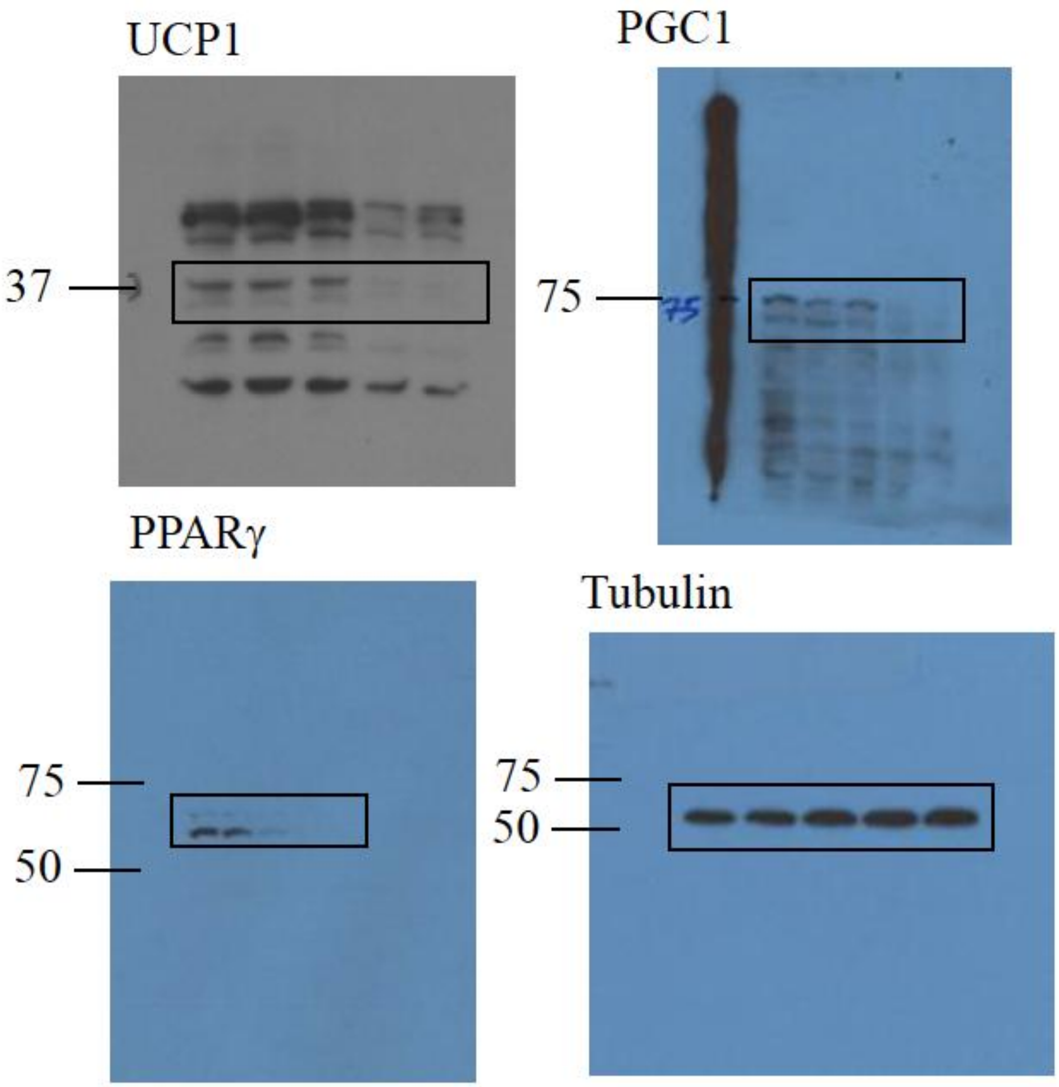

Figure S4. Uncropped images of Figure 1D.

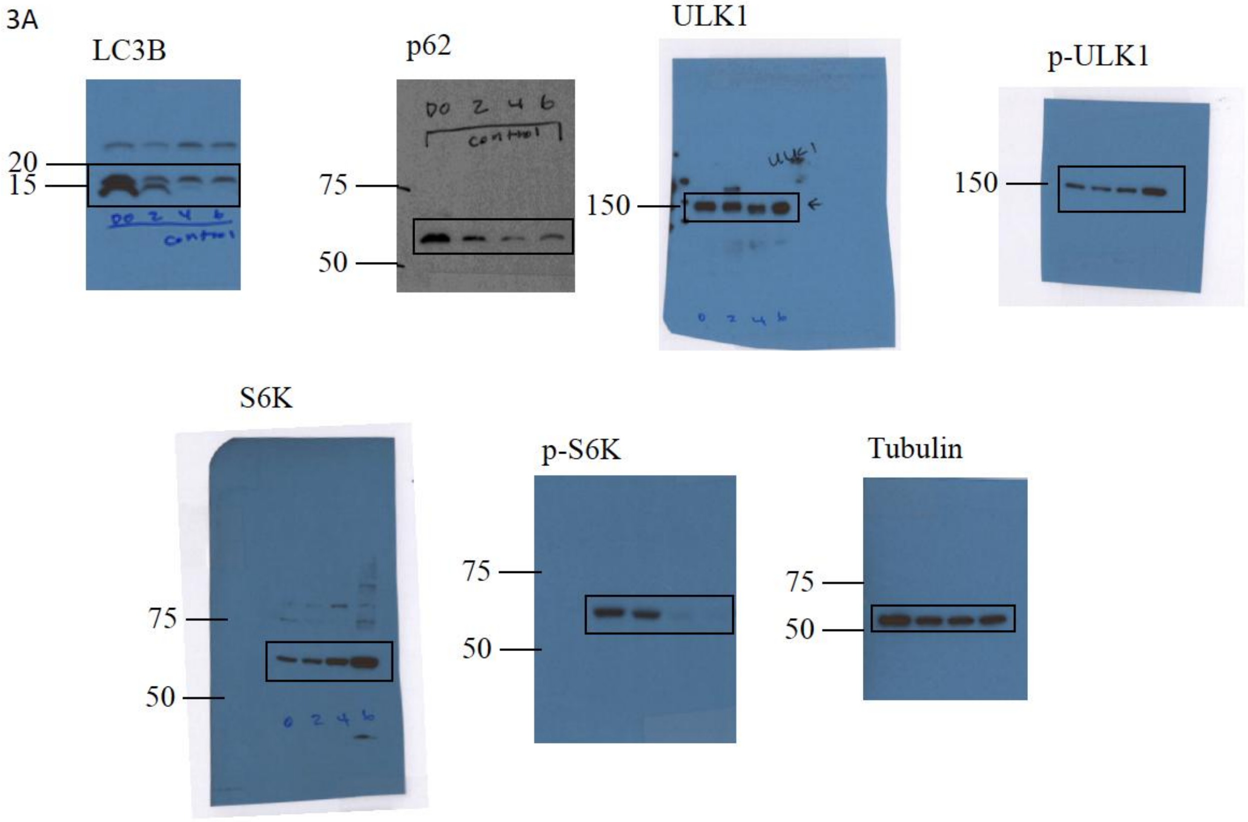

Figure S5. **Uncropped images of Figure 3A.**

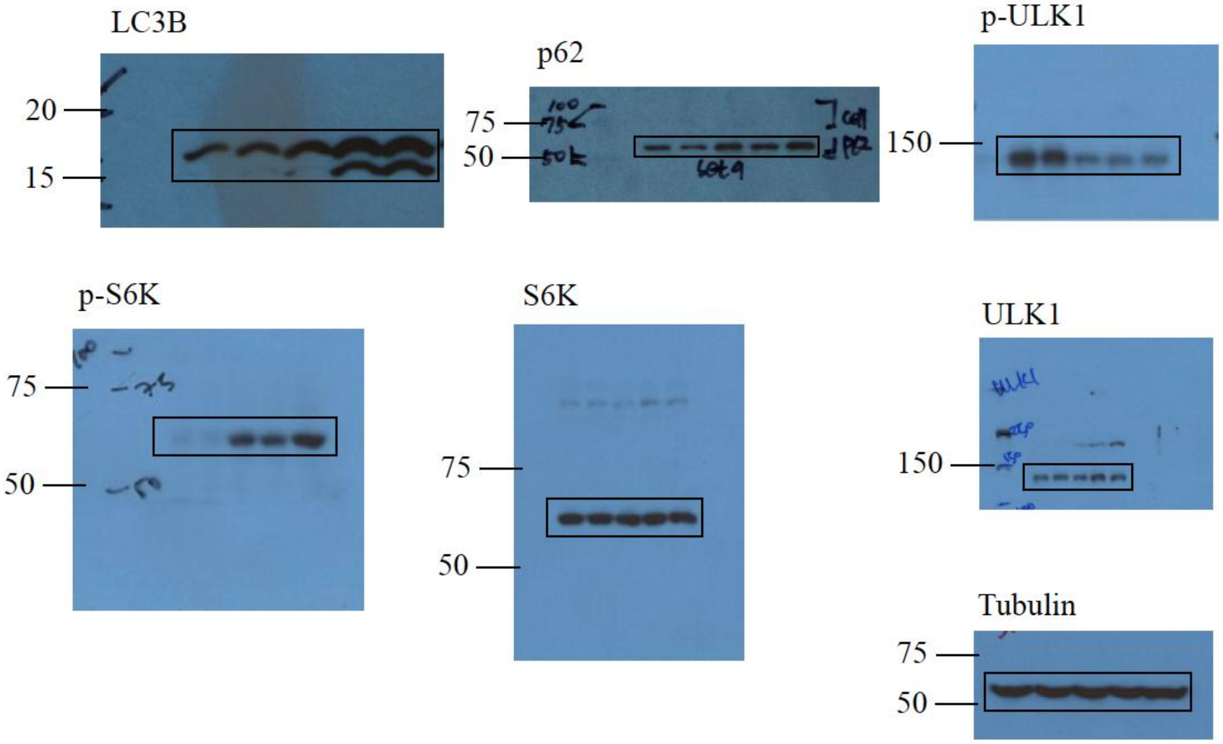

Figure S6. **Uncropped images of Figure 3B.**

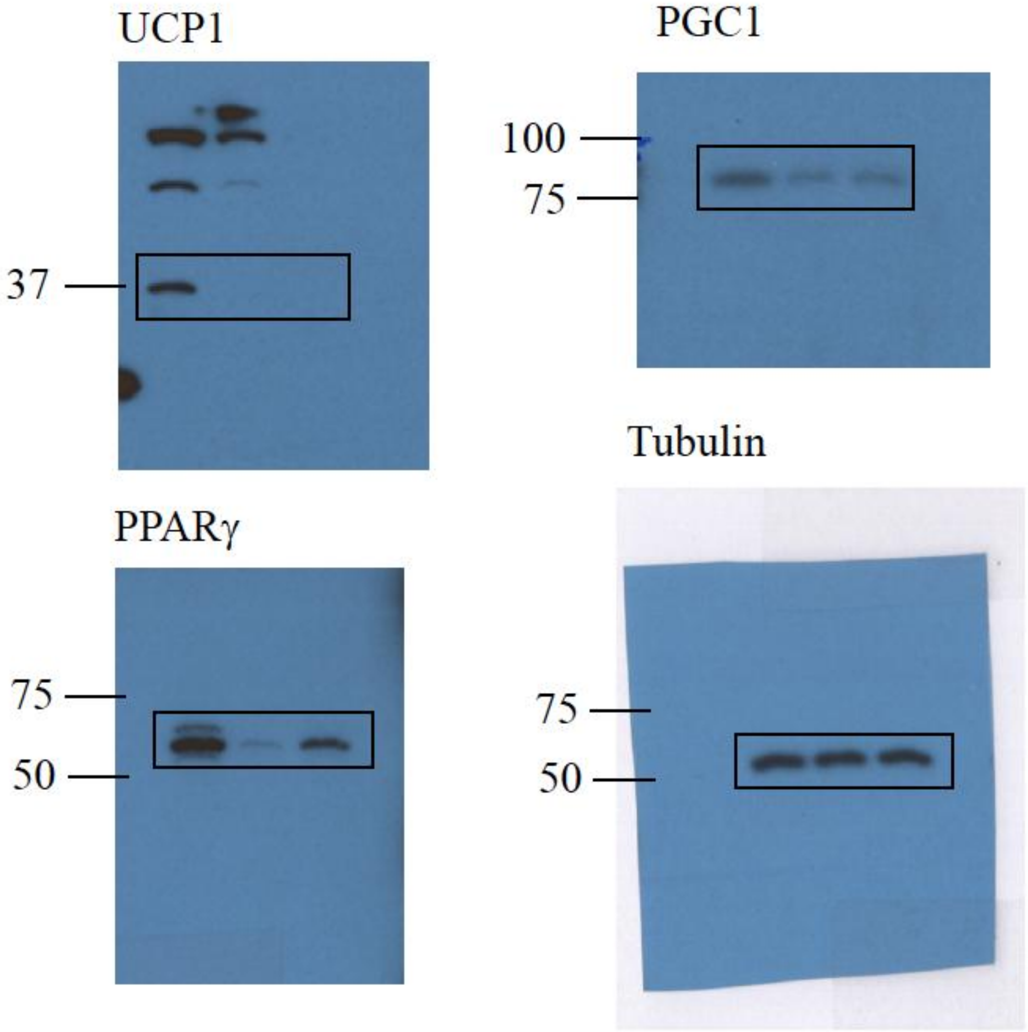

Figure S7. **Uncropped images of Figure 3C.**

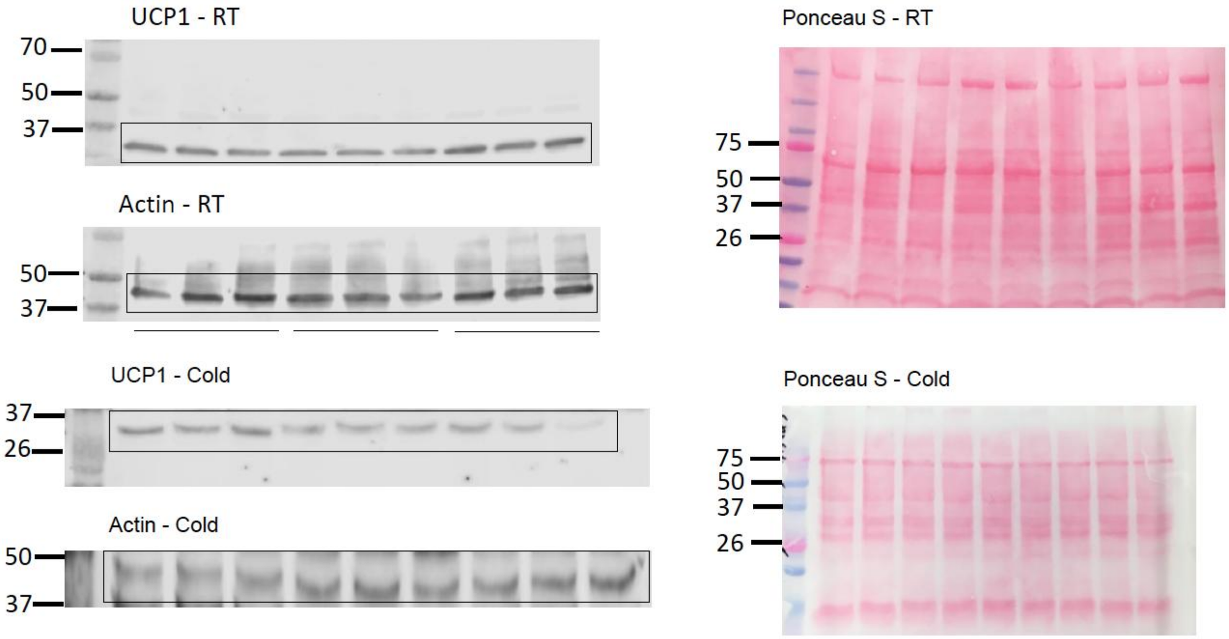

Figure S8. **Uncropped images of Figure 5C.**

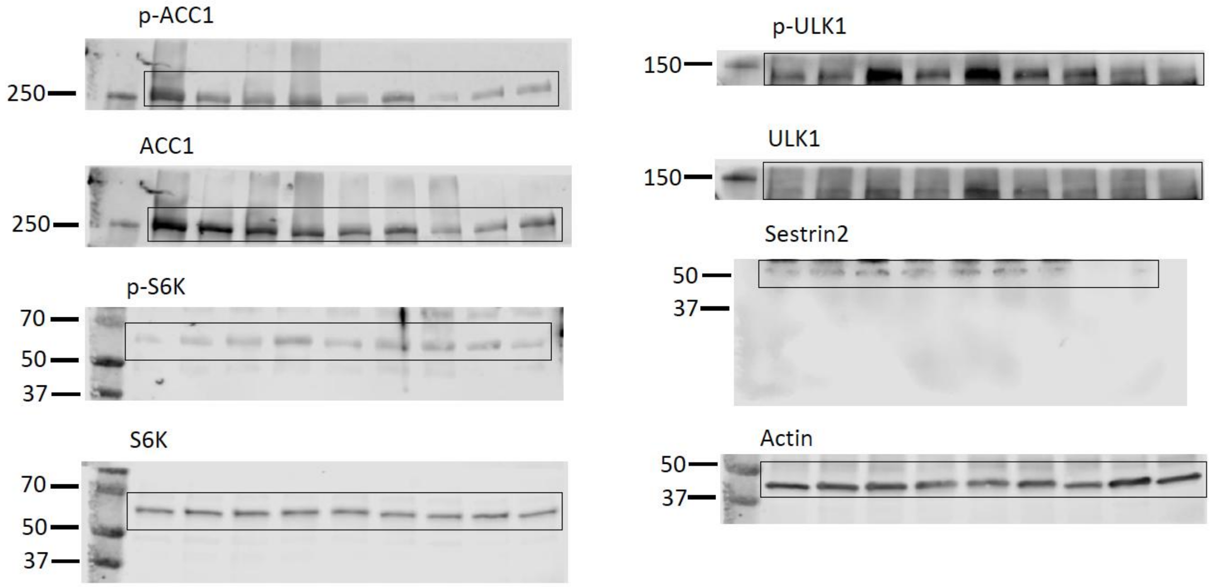

Figure S9. **Uncropped images of Figure 6B.**

Gene	Forward	Reverse
UCP1	GCTTTGCCTCACTCAGGATTGG	CCAATGAACACTGCCACACCTC
PGC1	GAATCAAGCCACTACAGACACCG	CATCCCTCTTGAGCCTTTCGTG
PPARγ	GTACTGTCGGTTTCAGAAGTGCC	ATCTCCGCCAACAGCTTCTCCT
PRDM16	ATCCACAGCACGGTGAAGCCAT	ACATCTGCCCACAGTCCTTGCA
Cox4a	TCATTGGCTTCACTGCGCTCGT	TCCAGCATTCGCTTGGTCTGCA
Uqcrh	GTGAGAGAGCACTGTGAACAGC	CAATCCTCTTCTGTCTGTGACCG
Tfam	GAGGCAAAGGATGATTCGGCTC	CGAATCCTATCATCTTTAGCAAGC
Nrf-1	CGAAAGAGACAGCAGACACG	TTGAAGACAGGGTTGGGTTT
Sestrin2	TAGCCTGCAGCCTCACCTAT	TATCTGATGCCAAAGACGCA
ULK1	GCACAGACAGCCTACAGGAGAAAC	GGTGGGGAGCCTACAGTAAATACC
Atg5	CCCCTGAAGATGGAGAGAAGAG	TCCTGACTCAAGGTGGTTCC
Atg7	ATGCCAGGACACCCTGTGAACTTC	ACATCATTGCAGAAGTAGCAGCCA
p62	ATCGGAGGATCCGAGTGT	TGGCTGTGAGCTGCTCTT
LC3B	CGGAGCTTTGAACAAAGAGTG	TCTCTCACTCTCGTACACTTC
Actin	CAAAAGCCACCCCCACTCCTAAGA	GCCCTGGCTGCCTCAACACCTC

Table S1. List of primer sequences used for real time RT-PCR.
